# Understanding Self-Controlled Motor Learning Protocols through the Self-Determination Theory

**DOI:** 10.3389/fpsyg.2012.00611

**Published:** 2013-01-11

**Authors:** Elizabeth A. Sanli, Jae T. Patterson, Steven R. Bray, Timothy D. Lee

**Affiliations:** ^1^Department of Kinesiology, McMaster UniversityHamilton, ON, Canada; ^2^Department of Kinesiology, Brock UniversitySt. Catharines, ON, Canada

**Keywords:** self-control, practice, feedback, motor tasks, motivation, autonomy support

## Abstract

The purpose of the present review was to provide a theoretical understanding of the learning advantages underlying a self-controlled practice context through the tenets of the self-determination theory (SDT). Three micro-theories within the macro-theory of SDT (Basic psychological needs theory, Cognitive Evaluation Theory, and Organismic Integration Theory) are used as a framework for examining the current self-controlled motor learning literature. A review of 26 peer-reviewed, empirical studies from the motor learning and medical training literature revealed an important limitation of the self-controlled research in motor learning: that the effects of motivation have been assumed rather than quantified. The SDT offers a basis from which to include measurements of motivation into explanations of changes in behavior. This review suggests that a self-controlled practice context can facilitate such factors as feelings of autonomy and competence of the learner, thereby supporting the psychological needs of the learner, leading to long term changes to behavior. Possible tools for the measurement of motivation and regulation in future studies are discussed. The SDT not only allows for a theoretical reinterpretation of the extant motor learning research supporting self-control as a learning variable, but also can help to better understand and measure the changes occurring between the practice environment and the observed behavioral outcomes.

## Introduction

### Self-controlled practice

There are many instances where individuals engage in movement activities, unprompted in order to try something new, challenge themselves on an already learned skill, or develop new skills. Ryan and Deci (2007, p. 2) describe this type of inherent inclination to engage in activities as intrinsic motivation. However, there are many additional aspects to practice, performance, and learning that can influence the individual and their behavior. For example, the characteristics of the environment where practice takes place can influence performance and learning as well as the quality of motivation experienced (see Lewthwaite and Wulf, 2012 for recent review). When it comes to learning motor skills, we often rely on the coach or teacher to organize the practice session and provide us with guidance as to how to practice. In the case of a basketball jump shot this may include the coach prescribing how many shots to take and from where, providing demonstrations of proper form and maybe providing feedback after some or all of the physical attempts. In this case, the practice context is defined by the coach (externally) rather than the learner themselves (termed self-controlled).

Challenging the athlete to achieve high levels of movement expertise in an externally defined practice context is commonly referred to as deliberate practice. Deliberate practice is defined by Ericsson et al. (1993) as being effortful, designed to improve performance, and not be inherently enjoyable. Ericsson et al. (1993) suggests that athletes engage in deliberate practice because they know it will improve their performance, at the expense of being a “fun” way to practice. Yet, would there be performance advantages if the performer retained some control over their practice context? Would practice become more fun and intrinsically motivating, or would it be burdensome? Would it positively or negatively affect learning?

In recent years, a number of studies in the motor learning domain have examined the advantages of providing the learner control over a portion of their practice context as a method of expediting skill acquisition. Collectively, the motor learning research suggests that providing choice to the learner during their practice positively impacts skill learning compared to when choice is not provided (Wulf, 2007). Learners have been provided the opportunity to control the following practice variables: the receipt of augmented feedback, including knowledge of results (KR; e.g., Patterson et al., 2011), knowledge of performance (KP; e.g., Patterson and Lee, 2010), concurrent feedback (e.g., Huet et al., 2009), the repetition order during multi-task learning (e.g., Keetch and Lee, 2007), and the amount of physical practice repetitions (e.g., Post et al., 2011). Other practice variables include controlling the frequency of observing a model or instructional video (e.g., Brydges et al., 2010) and the use of an assistive device (e.g., Hartman, 2007). The results from the aforementioned experiments suggest that providing the learner with control over a specific practice variable is a robust practice characteristic that facilitates motor skill acquisition. Although these findings appear conclusive, a theoretical understanding of the mechanisms underlying these advantages has remained elusive. Therefore, our purpose for the present review is to provide a theoretical interpretation of the motor learning advantages underlying a self-controlled practice context through the tenets of the self-determination theory (SDT).

### Self-determination theory

Self-determination theory is a macro-theory comprised of several micro-theories that can inform predictions made in self-controlled motor learning studies. Ryan and Deci (2007, p. 7) discussed three of these micro-theories in relation to sport and exercise and we have further applied them to a motor learning, self-controlled practice context.

**Figure 1:**
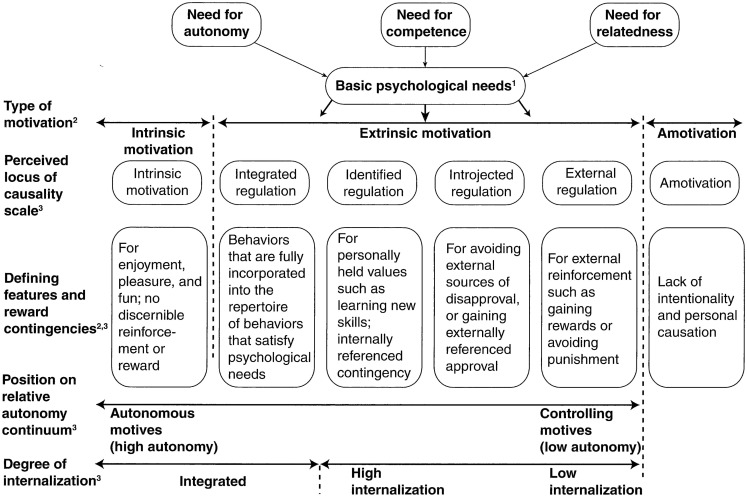
**Schematic representation of self-determination theory illustrating the features of three of the component subtheories: basic psychological needs theory, cognitive evaluation theory, and organismic integration theory**. ©Martin S. Hagger. Reprinted, with permission, from R.M. Ryan and E.L. Deci, 2007, Active human nature: Self-determination theory and the promotion and maintenance of sport, exercise, and health. In Intrinsic motivation and self-determination in exercise and sport, edited by M.S. Hagger and N.L.D. Chatzisarantis (Champaign, IL: Human Kinetics), 8.

The first of the micro-theories presented in Figure 1 (Basic psychological needs theory) addresses the three basic psychological needs of autonomy, competence, and relatedness which can influence the quality of motivation experienced by an individual. Autonomy involves feelings of willingness and choice in regards to activities undertaken; relatedness refers to feelings of closeness to other people; and competence involves feeling able to master challenges and having effective interactions with the environment (Katz and Assor, 2007). The quality of motivation is *enhanced* when any of these needs is satisfied and *optimized* if all three are satisfied. This micro-theory provides an illustration of the beginning of the motivational process and can illuminate individual differences in how well each of the needs are satisfied within a given practice context (Ryan and Deci, 2007, p. 7).

In the motor learning literature examining self-control, satisfaction of the psychological needs has not been explicitly examined, although they may have been influenced by features of the skill acquisition practice environment. As we will illustrate below, environmental or procedural supports for autonomy, competence, and relatedness may be included within the design of the practice contexts used in motor learning studies. Some designs may also include characteristics that could be detrimental to the satisfaction of the psychological needs for autonomy, competence, and relatedness.

The second micro-theory presented in Figure 1 is the Cognitive Evaluation Theory which describes circumstances within the person and the environment that can lead to behavior that is intrinsically or extrinsically motivated. If the behaviors undertaken by participants are *intrinsically motivated*, an activity will be performed out of interest, enjoyment, and/or satisfaction, where the purpose of the activity is the activity itself without the influence of consequences or threats of external or internal origin (Deci et al., 1996). In contrast, behavior can be extrinsically motivated, in which case the activity is performed with the intention of supporting personally held values, avoiding guilt, obtaining approval, or a reward or avoiding punishment (Deci et al., 1996). As mentioned earlier, deliberate practice is undertaken as a means to improve performance, rather than for purely intrinsic reasons. According to Ericsson’s definition, deliberate practice would be an example of an *extrinsically motivated* behavior.

The third micro-theory is the Organismic Integration Theory, which postulates that extrinsic motivation can be further divided across a continuum of four subtypes of behavioral regulation. At one end of the scale is *external regulation* which represents activities controlled by external demands or contingencies such as rewards or punishments (Deci et al., 1996). *Introjection* represents activities controlled by internal demands or contingencies such as guilt or embarrassment (Deci et al., 1996). Behaviors that are regulated by introjections are more likely to be maintained than externally regulated behaviors, but are still relatively unstable in terms of maintenance (Deci and Ryan, 2000). *Identified regulation* represents activities chosen because the person identifies with the importance of the activity and it may be important to achieve self-selected goals. Activities that are regulated by indentified regulation are associated with increases in commitment, performance, and maintenance, compared to those discussed above (Deci and Ryan, 2000). Closest to intrinsic motivation is *integrated regulation*, which is represented by activities that are experienced freely because they have been integrated within the person’s sense of self. The difference between intrinsic motivation and integrated regulation is that integrated regulation is performed freely because it is important to an important outcome and not for the sake of the activity itself (Deci et al., 1996). These different types of motivation fall along a continuum of feeling of ownership of the behavior. In other words, the amount of *self-determined motivation* increases in moving from external- to introjected- to identified- and finally integrated-behavior (Katartzi and Vlachopoulos, 2011). According to the SDT, the process of internalizing motivations occurs when moving along the continuum from external and controlling to ones that are more autonomous (Katartzi and Vlachopoulos, 2011). By definition, deliberate practice is extrinsically motivated, but factors within the environment, such as supports for autonomy could influence self-determined motivation to align closer to indentified regulation. Thus, the reasons participants engage in the behavior requested of them falls somewhere along the continuum from external to integrated regulation. The practice environment during acquisition, including the provision of choice such as in a self-controlled practice environment can influence where on the continuum any one participant may fall by either facilitating or inhibiting internalization in the learning process.

The SDT can be used to make predictions regarding motor learning within a particular practice protocol. The practice environment can be structured to provide varying levels of support for the satisfaction of the need for the three basic psychological needs, which subsequently can affect self-determined motivation and behavior. The consequences of internalization (or lack thereof) may be evaluated by looking at changes in cognition (concentration), affect, and behavior (Katartzi and Vlachopoulos, 2011). Studies in the motor learning domain infer persistent changes to motor behavior from measures such as movement time (e.g., Patterson et al., 2011), movement accuracy (e.g., Wrisberg and Pein, 2002), and movement form (e.g., Bund and Wiemeyer, 2004). In contrast, studies from the self-regulated learning literature examining changes in the social environment infer changes in self-determined motivation using such measures as engagement (e.g., Reeve et al., 2004), autonomous or intrinsic motivation (e.g., Vansteenkiste et al., 2004), and positive affect (e.g., Joussemet et al., 2004). One factor suggested to underlie the learning advantages in a self-controlled practice motor learning context is the increased motivation of the learner to adhere to the task goal. Despite the importance of motivation in facilitating motor skill learning (see Lewthwaite and Wulf, 2012 for review), a limitation of the motor learning research examining the benefits of a self-controlled practice context is that heightened motivation underlying the learning advantages has been assumed rather than directly measured and quantified.

The purpose of the present review is to offer an updated theoretical interpretation of the learning advantages commonly demonstrated in practice contexts providing the learner control over a portion of their practice context. We reviewed 26 peer-reviewed, empirical studies from the motor learning and medical training literature (requiring learning a motor skill), examining the learning benefits associated with the learner controlling at least one practice variable. Though several published abstracts were identified as relevant, they were not included in this review based on the limited information regarding the methodology. As well, studies that included clinical populations were also excluded. The focus of this review will be to: (1) examine and make explicit links from the body of motor learning literature reviewed examining self-control to each of the three micro-theories of the SDT, and (2) present explicit links between the SDT and the self-controlled practice contexts used to facilitate motor learning.

## The Environment during Acquisition

### Supports for autonomy, competence, and relatedness in the practice environment

Su and Reeve (2011) operationally defined five interpersonal conditions of autonomy support (based on Deci et al., 1994; Williams et al., 1999; Reeve et al., 2004) which can be identified within the motor learning protocols as a method of describing the psychological aspects of the environment during practice. Consistent across the reviewed motor learning experiments using self-control is the provision of *choice*, the most relevant of the conditions identified by Su and Reeve (2011). The choice provided to a learner over a specific practice variable (e.g., KR, assistive device, repetition schedule, etc.) is the common manipulation in the reviewed motor learning research; Table 1 provides an overview of key features of the practice environment for the papers reviewed. The use of a yoked condition in the motor learning research provides a method of distinguishing between the cognitive or motivational processes underlying the learning advantages of self-control, or the frequency at which the motor learning variable was received as the mechanisms responsible for learning. The yoked condition replicates the structure of the practice context individualized by a self-controlled counterpart, yet without the choice. This practice context resembles a *controlling* (yoked) verses *autonomy supportive* (self-controlled) environment as outlined by the SDT. The yoked group is not offered choice within the protocol and thus the protocol could be viewed as controlling because it decreases the opportunity for a person to experience a sense of autonomy.

**Table 1:** **Elements of self-controlled practice environment**.

	Motor Task Practiced	Practice variable controlled	Dependent Variables	Experimental groups	Temporal organization of experimental protocol
Andrieux et al. (2012)	Computer-based target interception	Task difficulty	CE distance between center of racquet and target and indication if target interception was early or late	Self-control* Yoked	A completed on 1 day IR 15 min post acquisition DR 24 h post acquisition
			VE distances from the target		
			AE difference between target and end position		
			Number of intercepted targets	
Brydges et al. (2010)	Intravenous catheterization using simulators	Practice schedule	Measures of technical skills (GRS and CL)	Open-ended*	A completed on 1 day	
			Global clinical performance (IPPI rating tool)	Progressive*	IPT
			Time spent on each simulator Time spent and exact portions of video viewing	Proficiency-based	DT 1 week post acquisition
				Yoked control	
			Total number of trials on each simulator	
			Rating of educational value of each simulator	
Brydges et al. (2009)	Wound closure skills	Access to video	Hand motion efficiency (time on task and total number of hand movements) Measure of technical skills (GRS and CL)	Self-process* Control-process Self-outcome* Control-outcome	A completed on 1 day DR 1 week post acquisition DT 1 week post acquisition
Bund and Wiemeyer (2004)	Forehand topspin table tennis stroke	Access to video Practice schedule	Accuracy score Form score based on a number of criteria assessed by independent raters	SC+* SC−* YO+ YO−	A completed on 1 day IR 5 min post acquisition DR 24 h post acquisition
Chen et al. (2002)	Timed key-pressing	Augmented feedback	VE variability in the difference between goal and actual times |CE| difference between goal time and actual performance	SI-KR* EI-KR* SI-yoked EI-yoked	A Completed on 1 day IR 5 min post acquisition DR 48 h post acquisition
Chiviacowsky and Wulf (2002)	Key-pressing with absolute and segmental goal times	Augmented feedback	AE difference in time between goals and actual performance (both relative and overall timing)	Self* Yoked	A completed on 1 day DR 24 h post acquisition DT 24 h post acquisition
Chiviacowsky and Wulf (2005)	Key-pressing with absolute and segmental goal times	Augmented feedback	AE difference in time between goals and actual performance (both relative and overall timing)	Self-after* Self-before*	A completed on 1 day DR 24 h post acquisition DT 24 h post acquisition
Chiviacowsky et al. (2008a)	No-vision, beanbag toss	Augmented feedback	Accuracy score	Self-control* Yoked	A completed on 1 day DR 24 h post acquisition
Chiviacowsky et al. (2008b)	No-vision, beanbag toss	Augmented feedback	Accuracy score	More KR* Less KR*	A completed on 1 day DR 24 h post acquisition
Hansen et al. (2011)	Timed key-pressing	Augmented feedback	Number of error trials CE amount of time and whether attempt was too fast or too slow VE variability in the difference between goal and actual times |CE| difference between goal time and actual performance	SC* TY YSC*	A completed on 1 day DR 24 h post acquisition DT 24 h post acquisition
Hartman (2007)	Stabilometer	Use of assistive device	Time in balance	Self-control* Yoked	A practiced on two consecutive days DR 24 h post acquisition
Hodges et al. (2011)	Right-handed backhand	Practice schedule	3D RE score distance between landing and target	Expert_Self-schedule*	A completed on 1 day DR 48 h post acquisition
	Left-handed backhand	Access to video	Movement form score from an eight-point scale	Novice_Self-schedule*	
	Right-handed forehand flying disk throws	Access to augmented information		Novice_Expert-schedule	
Jowett et al. (2007)	One-handed square knot	Access to video Practice schedule	Expert global rating scale assessments Economy of hand movements (total time and number of hand movements and path length)	Additional practice group* No additional practice group*	A completed on 1 day DR 1 week post acquisition
Huet et al. (2009)	Walk through a virtual corridor and pass through open doorways at the correct aperture	Augmented feedback	Variability of walking speed Success rate Variability in current error Variability in current error at time of FB request	Control Gage* Ghost doors* Yoked	A practiced over 4 days PT on all 4 days of acquisition DR 24 h post acquisition
Janelle et al. (1997)	Tennis ball throw with non-dominant hand	Augmented feedback	MRE distance between landing and target SRE distance between landing and target BVE variability in distance between landing and target Developmental stage of throwing Throwing speed	KR only Summary KP Self-controlled KP* Yoked control	A completed on 2 days, separated by 2 days DR 4 days post acquisition DT 4 days post acquisition
Janelle et al. (1995)	Underhand golf ball toss	Augmented feedback	AE difference between target and end position of trials	Performance – summary	A Completed on 1 day IR 10 min post acquisition
				Fifty percent	
				Subject-controlled*	
				Yoked control	
				Control	
Keetch and Lee (2007)	Sequence of aim – and-click movements with a computer mouse	Practice schedule	Pattern error (incorrect button press) Cursor error MT	Hard-random Hard-blocked Easy-random Easy-blocked Hard-yoked Hard-self-regulated* Easy-yoked Easy-self-regulated*	A completed on 1 day DR 24 h post acquisition
Patterson and Carter (2010)	Key-pressing with absolute and segmental goal times	Augmented feedback	% |CE| difference between goal time and actual performance	Self-regulated* Yoked	A completed on 1 day IR 15 min post acquisition DR 24 h post acquisition DT 24 h post acquisition
Patterson et al. (2011)	Timed key-pressing	Augmented feedback	VE variability in the difference between goal and actual times |CE| difference between goal time and actual performance	Self-self* All-self* Faded-self* Yoke-yoke All-yoke Faded-yoke	A completed on 1 day IR 10 min post acquisition DR 24 h post acquisition DT 24 h post acquisition
Patterson and Lee (2010)	Production of PDA symbols matching English cues	Access to augmented information	Recall	Proactive self-regulated* Proactive yoked	A completed on 1 day IR 10 min post acquisition DR 48 h post acquisition
				Proactive every trial	
				Retroactive self-regulated*	
				Retroactive yoked	
				Retroactive every trial	
Post et al. (2011)	Dart throw with non-dominant hand	Practice schedule	VE variability in distance from the target RE distance from innermost target to the tip of the dart Average preparation time Recall of number of trials completed	Self-control* Yoked	A completed on 1 day DR 24 h post acquisition DT 24 h post acquisition
Wrisberg and Pein (2002)	Badminton long serve	Access to video	Accuracy score	ALL	A practiced over 3 days
			Expert scores for five task-specific components	LC* NM	DR 24 h post acquisition
Wu and Magill (2011)	Key-pressing with relative time sequences	Practice schedule	AE deviation of performance from goal time	Self-control*	A completed on 1 day
			E overall performance accuracy taking response bias and variability into account	Yoked	IT 5 min post acquisition DT 24 h post acquisition
			RTE		
Wulf et al. (2001)	Ski simulator	Use of assistive device	Amplitude Frequency of movements Relative force onset of movements	Self-control* Yoked	A practiced on two consecutive days DR 24 h post acquisition
Wulf et al. (2005)	Basketball jump shot	Access to video	Accuracy score Movement quality score from six task-specific criteria	Self-control* Yoked	A completed on 1 day DT 1 week post acquisition
Wulf and Toole (1999)	Ski simulator	Use of assistive device	Amplitude	Self-control* Yoked	A practiced on two consecutive days DR 24 h post acquisition

*Denotes groups that controlled the practice variable; CE, constant error; VE, variable error; AE, absolute error; GRS, Global Rating Scale; CL, the procedural checklist; IPPI, integrated procedural performance instrument; LC, learner controlled; NM, never model; |CE|, absolute constant error; 3D RE, three dimensional radial error; FB, feedback; MRE, mean radial error; SRE, subject-centroid radial error; BVE, bivariate radial error; MT, movement time; RE, radial error; E, total variability; RTE, relative timing error; A, acquisition; IR, immediate retention; DR, delayed retention; IPT, immediate post test; DT, delayed transfer; PT, post test; IT, immediate transfer.

One benefit of allowing participants control over at least one aspect of their practice environment is the opportunity for the learner to tailor their practice to their own individual needs and capabilities (Wulf, 2007). For example, participants choosing to use ski poles to facilitate their motor performance on every practice attempt during an acquisition session would be considered a less challenging environment than if they never asked for the ski poles or if they gradually faded the requests across the acquisition period. The opportunity for the learner to adjust their practice environment as a method of optimally challenging the cognitive and motor processes of the learner provides support for the basic need for feelings of competence as well as autonomy as outlined by Su and Reeve (2011).

The other four interpersonal conditions identified by Su and Reeve (2011) as having an impact on feelings of autonomy are; (1) the provision of a meaningful rationale, (2) acknowledgment of feelings that may be negative, (3) attempts to nurture inner motivational resources, and (4) the use of non-controlling language. The provision of a meaningful rationale or explanations as to why the activity would be useful to the learner can facilitate the learners’ understanding of why they are being requested to complete the task (Su and Reeve, 2011). Acknowledging that what is being requested of the learner may not be desirable and that any feelings of conflict are legitimate can also support feelings of autonomy (Su and Reeve, 2011). Though, no specific instances of acknowledging negative feelings were reported in the reviewed motor learning literature, it is impossible to rule out that feelings of fatigue or boredom may have occurred in participants, especially in the yoked or control conditions where they were not encouraged to be actively involved in their learning, and as a result, demonstrated inferior learning to their self-controlled counterparts. Attempts to nurture inner motivational resources are described by Su and Reeve (2011) as the vitalization of the learners’ enjoyment, needs satisfaction, or sense of challenge or curiosity, during the activity. In other words, explicit attempts to satisfy the need for autonomy, competence, or relatedness can be found (though rarely) in the motor learning, self-controlled literature. The use of non-controlling language means the avoidance of words such as “should,” “must,” and “have to” to convey a sense of choice or flexibility (Su and Reeve, 2011). Although, the specific scripts or instructions are often not included in the methodologies of the motor learning experiments reviewed, some examples of both non-controlling and controlling language were identified. For example, Brydges et al. (2010) told participants that “if you feel that you have learned the task proficiently, you do not need to stay the full 2 h,” which could be viewed as non-controlling whereas Bund and Wiemeyer (2004) and Janelle et al. (1997) told participants to focus on movement form or mechanics rather than outcome, which could be viewed as more controlling.

In summary, we can, see that there are many opportunities to influence the amount of support for feelings of autonomy, competence, and relatedness within a motor learning protocol. Presently in the motor learning, self-controlled literature, some examples of both supporting and thwarting factors can be identified. In the future, explicit attempts to address the support of the three basic needs in the design, execution, and reporting of experiments would provide a more complete picture of the influence of motivation on learning of a motor skill.

In the following sections of this chapter, instances of these conditions and other indications of support for the three basic needs central to the basic psychological needs theory will be discussed in the context of protocol design.

### Control of augmented feedback

The most commonly manipulated aspect of the learning environment is the scheduling of augmented feedback. The learner has been provided the opportunity to control three types of feedback in the motor learning literature examining self-control. KR informs the learner about the outcome of their motor action compared to the goal, whereas KP provides information to the learner regarding the technical aspects (e.g., movement form). Both KR and KP are provided to the learner *after* the motor task has been completed (Schmidt and Lee, 2011, p. 394). Concurrent feedback on the other hand, provides information to the learner in regards to their approximation of the task goal *during* the performance of the motor task (Schmidt and Lee, 2011, p. 394).

A number of studies have provided participants the opportunity to request KR (e.g., Chen et al., 2002) or KP (e.g., Janelle et al., 1997) after the completion of each trial or after a pre-determined number of trials. Huet et al. (2009) provided learners the opportunity to request concurrent feedback during acquisition trials, to the advantage of learning. The motor tasks examined in the aforementioned studies have ranged from fine-motor key-pressing tasks with specified timing goals (Chen et al., 2002) to gross motor tasks such as a ball toss (Janelle et al., 1995, 1997) and a virtual reality task (Huet et al., 2009).

In addition to a group provided with self-control, at least one yoked control group was included in the experiments examining self-controlled feedback. Participants in these groups replicated the augmented feedback of that chosen by a self-controlled counterpart, but without the choice. In some experiments, additional experimental or control groups were incorporated to examine the utility of a self-controlled context. For example, Janelle et al. (1995) included control groups with feedback provided for varying percentages of the total number of acquisition trials, in addition to the self-controlled feedback and yoked conditions as a method of examining the influence of the absolute amount of KP and self-control. In a follow-up experiment, Janelle et al. (1997) expanded the control groups used by Janelle et al. (1995) to include one group that received no augmented information during acquisition. Chen et al. (2002) included a self-controlled KR condition and an experimenter-induced KR condition along with their respective yoked counterparts to examine the effects of differing levels of autonomy in the choice of whether or not to receive KR. In another example, Patterson et al. (2011) manipulated the amount of KR provided in the first half of acquisition (all trials, a faded schedule, or a self-controlled schedule), prior to a period of self-controlled KR. A yoked group was also included for each of those conditions. Hansen et al. (2011) included a self-controlled KR group and two different yoked groups. The first yoked group replicated the KR schedule of a self-controlled counterpart (e.g., traditional yoked condition), whereas the second yoked group were provided an absolute number of KR trials, based on the number of KR trials requested by the self-controlled counterpart, and were subsequently provided the opportunity to request KR based on their provisional limit. The experimental groups differed in the cognitive demands placed on the learner. Those in the yoked condition with control over their receipt of KR had fewer opportunities to request KR and experienced having higher cognitive demands compared to the traditional yoked or self-controlled group. According to the SDT, the yoked group provided choice over the number of times and the timing of feedback would be expected to experience a greater feeling of autonomy than those given choice over their receipt of KR on all acquisition trials. In attempts to examine factors that modulate the learning advantages of a self-controlled KR context, Chiviacowsky and Wulf (2005) asked learners whether or not they required KR in one of two conditions, either before or after the trial. Chiviacowsky et al. (2008b) recently examined whether usefulness of a self-controlled KR context for children was based on the proportion of trials for which KR was requested. Though in these instances the experimental groups were all provided choice, a support for feelings of autonomy, other factors differing between groups may have had an influence on motivation. For example, those who were able to choose to receive KR after a trial rather than making the choice prior to an attempt, could use a request for KR after what they felt to be a good attempt as a way to support feelings of competence while the group that chose prior could not.

Examples of meaningful rationales are limited within the motor learning protocols examining self-control. However, Janelle et al. (1997) explained to learners that they would learn to throw better through improved form rather than just focusing on outcome. In many studies examining a self-controlled practice schedules, participants were told to request task information *only when necessary* as a method of increasing the meaningfulness of the task related information (e.g., Chiviacowsky and Wulf, 2002). Several of the studies specifically indicated that participants were told that they would later be tested *without* the use of the practice variable that they were able to control during the acquisition period (e.g., Chiviacowsky and Wulf, 2002). Such instructions to the learners suggest a rationale for practicing at least some of the time without the requested practice variable. The encouragement of participants *to do their best* by Janelle et al. (1995) is an instance of nurturing inner motivational resources. The opportunity to request feedback in order to confirm a good trial or to correct a poor trial may have differential effects on feelings of competence, subsequently providing the opportunity to nurture inner motivational resources.

Some of the language used in the reviewed motor learning literature could be considered controlling rather than autonomy supportive. For example, Janelle et al. (1997) told participants to focus on movement form or mechanics rather than outcome. Chiviacowsky and Wulf (2002, 2005) told their participants in the self-controlled group that they “had to” control feedback frequency. Some self-control opportunities presented to learners came with qualifiers such as “request feedback only when you think you need it” (e.g., Chiviacowsky and Wulf, 2002) or “request feedback on 3 of 10 trials in each block” (Chiviacowsky and Wulf, 2005). These qualifiers may have been viewed as controlling and therefore would detract from the feeling of autonomy. Further, protocols that provide an opportunity for the learner to control one or more aspects of augmented feedback have been manipulated in various ways that either provide more or less support for feelings of both autonomy and competence.

### Control of access to video or augmented information

Studies such as Wrisberg and Pein (2002) provided the learner the opportunity to control when to view a videotaped demonstration of the to-be-learned motor task. The studies providing control over access to a video demonstration used gross motor sport skills such as a badminton serve, a table tennis stroke, and basketball jump shot.

Brydges et al. (2009) provided learners with access to specific instructions in regards to completion of a fine-motor surgical suturing skill while Patterson and Lee (2010) required learners to produce novel cursive handwriting characters while being provided the opportunity to view a visual display of the required character either before or after the required motor action. In most cases, at least one yoked control group was included, which followed an augmented information or viewing of a video schedule identical to one chosen by a self-controlled schedule counterpart, but without the choice. Wrisberg and Pein (2002) did not make use of a yoked condition, but instead used a control group that viewed the model on all trials and another control group that viewed the model on none of the acquisition trials. Both control groups in Wrisberg and Pein’s (2002) study were in situations that could be considered more controlling than the self-controlled group. One group may have had to watch a model when they did not want to while the other group may have wanted to view a model but were unable to. In both cases participants may have felt they were in a controlling environment. Bund and Wiemeyer (2004), Brydges et al. (2009), and Patterson and Lee (2010) each made use of two different self-controlled conditions with respective yoked counterparts. Differences in the satisfaction of the three basic needs may have occurred between self-control groups, despite the common autonomy supportive condition of the provision of choice. Bund and Wiemeyer (2004) provided one group with control over what was determined to be a preferred variable (viewing of a model) and another over a non-preferred variable (direction and length of serves). This manipulation addresses a possible difference in feelings of autonomy (preferred variable) and control (non-preferred variable). Brydges et al. (2009) yoked participants to the specific portions of the video viewed for both a process goal and an outcome goal subgroup. The manipulation of goal type may have created differences in the feelings of autonomy and competence. Patterson and Lee (2010) asked one group to decide whether or not to view the appropriate typographical symbol prior to the beginning of the trial and the other group after the trial was completed. Similar to Chiviacowsky and Wulf (2005) with KR, those that were able to choose to view the symbol after an attempt could choose to confirm a perceived good trial influencing feelings of competence, whereas those choosing prior to an attempt could not. These study designs allowed the examination of factors that modulated the usefulness of a self-controlled context.

Evidence for the provision of meaningful rationale can be seen in the explanation by Wulf et al. (2005) to participants that the video of the expert model performing a basketball jump shot could be used as a general reminder or for the observation of specific details. In another example, Bund and Wiemeyer (2004) stressed to participants that increased accuracy would result from correct form during practice, however focusing on movement form or mechanics rather than outcome suggests these instructions are controlling rather than autonomy supportive. As a more explicit example of providing a meaningful rationale, Brydges et al. (2009) provided participants with a list of goals for the to-be-learned motor task suggesting a rationale as to why the task should be performed in a certain way.

### Control of use of an assistive device

Wulf and Toole (1999), Wulf et al. (2001), and (Hartman, 2007) provided learners the opportunity to use an assistive device (ski poles) during performance of a ski simulator task or a pole for a stabilometer task. All three of the above studies used both a self-controlled use of the assistive device condition as well as a yoked condition. Wulf et al. (2001) had participants complete acquisition in self-controlled/yoked pairs in order to examine if the benefits of a self-controlled schedule would persist under dyad conditions, where motivational level may have been similar between the groups.

In all three studies discussed above, it was explained to participants that use of an assistive device to aid balance during acquisition could facilitate learning of a task and participants were told that they would later be tested *without* the use of the device. This information provided to the learners is suggested to resemble the provision of meaningful rationales for the use and scheduling of the assistive devices. In addition the study by Wulf et al. (2001) provides the only example to our knowledge in the current motor learning self-controlled literature where the satisfaction of feelings of relatedness may have come into play.

### Control of practice schedule and task difficulty

Learners have also been provided the opportunity to control the practice schedule itself. This includes the order of practicing multiple motor tasks during acquisition (e.g., Keetch and Lee, 2007) or the total number of physical trials to be completed (e.g., Post et al., 2011). Keetch and Lee (2007) compared self-controlled and yoked practice conditions to externally defined, blocked, and random practice schedules for both easy and hard versions of a motor task. Wu and Magill (2011) compared a self-controlled condition controlling the practice order of timing goals, and a respective yoked condition.

Andrieux et al. (2012) recently provided participants the opportunity to control the difficulty of the motor task. Manipulating task complexity was accomplished by asking participants to choose the racquet width to be used in an interception task. Andrieux et al. (2012) showed participants the most difficult version of the task at the beginning of acquisition and explained that it would be used in the later retention tests. This instance is an example of providing a meaningful rationale for the choice of task difficulty where the ability to choose task difficulty could appeal to the learner’s sense of challenge. In contrast, Post et al. (2011) provided a monetary incentive based upon performance in retention. However, introduction of an external reward such as money has been shown to be controlling, rather than supporting of an autonomous context (Joussemet et al., 2004).

### Control of multiple aspects of the practice environment

Three of the studies reviewed provided the learner the opportunity to control multiple aspects of the learning environment. For example, Brydges et al. (2010) allowed participants in one condition control over both the *timing of progression* from easier to more difficult versions of the task and *when to stop practice*. Those in a second condition were provided the freedom to move between all difficulties of the task as well as when to end practice. A yoked group as well as a proficiency-based progression group were also included. Brydges et al. (2010) required nursing students to learn an intravenous catheterization on a simulator. In another example, Jowett et al. (2007) provided all participants with unlimited access to a multimedia training video during acquisition of a novel surgical knot-tying task. Participants were also provided the opportunity to cease practice when they felt they had reached a proficient level of skill. Jowett et al. (2007) did not include a yoked group but rather split the self-controlled group into two conditions, one of which allowed participants to stop practice when requested, and those in the other condition were prescribed additional practice after the decision to stop had been made. Hodges et al. (2011) included two conditions where participants were given control over the number of attempts made, the order of trials when practicing the three disk throwing tasks, the amount of rest during practice, the frequency of access to verbal instructions, a video replay of the just-completed trial, and a video demonstration. Participants were also able to select which part of the attempt that they could receive instruction about. The two self-controlled groups differed in terms music playing expertise, however all participants were novices in the disk throwing tasks used in the experiment. A group yoked to the music experts was also included.

Similar to Brydges et al. (2009), Brydges et al. (2010) provided participants with a list of goals for the to-be-learned motor task suggesting a rationale as to why the task should be performed in a certain way. They also made use of process goals which might be considered nurturing to inner motivational resources. In the Brydges et al. (2010) protocol, participants were afforded the opportunity to directly manipulate the difficulty of the task for any given trial, by choosing when to progress to a higher fidelity (more difficult) simulator, appealing to the learners’ sense of challenge. Brydges et al. (2010) also provided a good example of non-controlling language telling participants “if you feel that you have learned the task proficiently, you do not need to stay the full 2 h.”

Jowett et al. (2007) included an example of differing levels of autonomy support between groups. They provided choice as to when participants believed they had reached a sufficient level of proficiency and could decide when to stop practice. One group did stop practice when requested, but another group was required to complete additional practice, which would undermine feelings of autonomy. Similar to Post et al. (2011), discussed above, Hodges et al. (2011) provided a monetary incentive based upon performance in retention, potentially undermining feelings of autonomy.

## Consequences of the Environmental Conditions during Acquisition

### Psychological measures

Though all the reviewed motor learning experimental protocols reported changes in behavior, only one study measured changes in concentration (mental effort) and affect (satisfaction). Hodges et al. (2011) found that a group of music experts that chose a schedule with frequent switching (high contextual interference) amongst motor tasks increased their satisfaction with practice more-so than those experts that switched less frequently or the novices that switched frequently. For the group of music experts, satisfaction and mental effort were correlated, but not for the novices.

Similarly, Bund and Wiemeyer (2004) measured self-efficacy throughout the experimental protocol and found that those in the self-controlled groups reported higher self-efficacy beliefs than those in the yoked groups. In particular they showed less of a decrease in self-efficacy beliefs after the first half of practice and higher efficacy expectations prior to each retention test.

### Behavioral measures

#### Measured variables

The measures of motivational consequences that have been used most often by motor learning researchers are measures of changes in behavior. The variety of dependent variables used in the papers reviewed is substantial and largely dependent upon the task (see Table 1). The most widely used class of dependent variables for measuring changes in behavior (learning) is that of error measures. The precision of measurement of error ranges from simple measures of accuracy (e.g., Bund and Wiemeyer, 2004) to very specific measures of the amount and direction of error (e.g., constant error measured by Hansen et al., 2011). Many of the studies reviewed used accuracy scores, often when referring to where a projectile has landed based upon preset targets (e.g., Wrisberg and Pein, 2002). In some cases the number (or average number) of trials for which an error was committed were reported (e.g., Hansen et al., 2011). Keetch and Lee (2007) reported both pattern error, which consisted of an incorrect button press, and cursor error which occurred when a button press took place when the cursor was not in the correct place. More specific measures of error used amongst the protocols reviewed are included in Table 1. Constant error measures the average response error and reports both magnitude and direction (Schmidt and Lee, 2011, p. 27), whereas variable error measures the inconsistency of the outcomes performed by the learner and compares participants’ outcomes to each other, without taking the goal into account (Schmidt and Lee, 2011, p. 28). Absolute error measures overall accuracy and reports the absolute difference between a target and the actual performance, disregarding direction (Schmidt and Lee, 2011, p. 27). The use of absolute constant error provides less misleading group results than CE. Some studies used two dimensional (e.g., Janelle et al., 1997) or three dimensional (e.g., Hodges et al., 2011) error scores for accuracy. Wu and Magill (2011) used RTE to measure the accuracy of the performance of relative timing across the entire trial.

In addition, motor learning researchers also use measures of movement quality. These include expert ratings and standardized rating scales (e.g., Brydges et al., 2009) as well as movement form or quality scores (e.g., Bund and Wiemeyer, 2004; Wulf et al., 2005). Descriptors of movement were included as dependent variables for a number of studies. For example, for a ski simulator task, both Wulf and Toole (1999) and Wulf et al. (2001) measured amplitude in centimeters. Measurements of movement time were also used as dependent variables such as by Keetch and Lee (2007) who measured the overall movement time for each trial. Included in some experiments were measures of memory recall of the required movement pattern (e.g., Patterson and Lee, 2010).

These specific dependent variables, used to measure changes in behavior, are usually the focus of motor learning self-controlled practice research, however, they are only one category of measures of the three identified by Katartzi and Vlachopoulos (2011) as being useful to describe changes due to motivation. Though measures of changes in behavior provide the most prominent way to measure the effects of experimental manipulations, the addition of measures of changes in affect and concentration in future experimental protocols would provide a more complete picture of the motivation consequences of manipulations.

Although the variety of dependent variables used to measure changes in motor behavior is vast, the learning benefits observed are remarkably consistent. Twenty-five of the 26 studies reviewed included one or more retention tests, while nine included a transfer test. Of those nine, eight included both retention and transfer. A retention test measures how well a task that was practiced during acquisition is retained, independent of the practice condition experienced during acquisition, whereas a transfer test measures how well the components learned during acquisition transfer to a novel version of the task (Schmidt and Lee, 2011, p. 462). Tests of learning, or relatively permanent changes in behavior can be either immediate or delayed. Immediate tests are performed shortly after the acquisition period on the same day. Delayed tests are performed after a longer period of non-practice, preferably after sleep has occurred (Walker et al., 2002).

#### Transfer

It has been suggested that a transfer test may be more sensitive than retention tests in capturing learning effects, as it requires participants to adapt to a novel context (Chiviacowsky and Wulf, 2002; Post et al., 2011). However the majority of papers reviewed did not include a transfer test. Some studies found a significant difference between groups only for transfer and not for retention; though in some cases retention was not measured. For example, Wu and Magill (2011) found that those afforded self-control during acquisition performed better than their yoked counterparts for both immediate and delayed transfer tests for all measured dependent variables. Chiviacowsky and Wulf (2005) found that participants provided choice as to whether or not to receive feedback prior to attempting the task performed with greater overall and relative timing error on a transfer test in comparison to those provided choice following each attempt.

Though Post et al. (2011) and Chiviacowsky and Wulf (2002) found benefits of a self-controlled practice context only in transfer, Brydges et al. (2009) found that the benefit of self-control was evident only in retention and not transfer. Patterson and Carter (2010) found that self-control over feedback provided benefits for performance measured during retention and for transfer tests (%|CE|). Brydges et al. (2010) found that both groups that were provided control over a portion of their practice maintained performance from the post test to the transfer test while those following experimenter-defined practice significantly decreased in performance, though the self-controlled groups did not maintain this benefit on a post test.

In summary, self-control motor learning studies thus far indicate that those provided with choice over at least one aspect of the practice environment, perform equally, or more often, better than those not provided with choice when asked to transfer skills to a novel task. A more autonomy supportive environment provides one possible explanation for this positive change in behavior, according to the SDT.

#### Retention

Along with the benefits found in transfer, Patterson and Carter (2010) found that self-control over feedback provided benefits for performance measured during retention tests (both % |CE| and CV) while Brydges et al. (2009) found that the benefit of self-control was evident only in retention and not transfer and was moderated by the type of goals set. Specifically, those who set *process* goals outperformed their yoked counterparts and those who set *outcome* goals did not outperform their yoked counterparts (Brydges et al., 2009).

In some studies, two or more experimental groups were provided with self-control, and in some cases with yoked counterparts or other experimenter-defined contexts. Patterson et al. (2011) found regardless of what percentage (50 or 100%) of the acquisition trials choice was provided or the type of KR schedule preceding the self-controlled KR portion, those provided self-control over KR outperformed yoked counterparts on retention tests (e.g., absolute constant error for all three self-controlled groups; variable error for two of the three self-control reaching statistical significance). Though measures of VE did not significantly differ between the self-control and yoked groups during transfer, the self-controlled groups demonstrated less |CE| than yoked groups with two of the three differences reaching statistical significance (Patterson et al., 2011). Hansen et al. (2011) found that those provided with an intermediate amount of self-control over their KR schedule (control over when to receive KR but yoked to the absolute number of times KR was provided) committed fewer errors on the retention test than those provided greater (control over schedule and number of times KR was provided) and lesser (schedule and amount of KR was yoked) amounts. Benefits of self-control were seen in transfer tests by Brydges et al. (2010), however, those under one of the experimenter-defined practice contexts performed best on the post test. These results indicate that providing nursing students the opportunity to choose which simulators to use was as effective as basing progressions on pre-defined proficiency criteria.

The benefits of self-control are still clearly evident in the results of the retention tests conducted. Janelle et al. (1995) measured immediate retention and found those in the self-controlled condition were more accurate on the retention test than the yoked and experimenter-controlled conditions. Andrieux et al. (2012) measured both immediate and delayed retention and found benefits of self-control over yoked groups in both tests.

Some studies using immediate and delayed retention tests compared two self-controlled practice conditions to each other as well as to yoked and control groups. Patterson and Lee (2010) found benefits for both immediate and delayed retention over yoked and control groups for those who self-controlled their receipt of augmented information, but only when given task related information prior to attempting the motor task. The distinction was not evident in those that were given the information after attempting the trial. The results of this experiment showed that information about “what to do” (e.g., proactive information) was just as beneficial as retroactive information, but only if the learner was provided control over the proactive information. Similar to the findings of the KR research, providing the learner control over receiving information about “what to do” had a positive impact on motor skill learning (e.g., Patterson and Lee, 2010). Chen et al. (2002) found that for immediate retention, both groups provided with self-control over KR performed with less |CE| than their yoked counterparts. This was also true for the delayed retention test with the addition of a significant difference between the two self-control groups.

Those that received a reminder of the choice provided to them outperformed those that did not receive the reminder on each trial (Chen et al., 2002). Bund and Wiemeyer (2004) found that regardless of whether choice was given in respect to a preferred or non-preferred element of practice, those who got to choose performed with better form than those in the respective yoked conditions on a delayed retention test. No differences between self-controlled and yoked nor preferred and non-preferred conditions were significant on the immediate retention test. Jowett et al. (2007) found no differences on post tests and delayed retention tests between those that received additional practice after choosing to stop practice and those that did not.

Chiviacowsky et al. (2008b) compared one group that chose to receive KR *frequently* when provided control and one group of participants that chose to receive KR *less frequently*. Participants who chose more frequent KR better maintained accuracy scores for the retention test and were significantly more accurate than those who had chosen less frequent KR. Hodges et al. (2011) measured the delayed retention results for one yoked and two self-controlled groups. Music experts who had many years managing practice of a skill unrelated to the one used in the experiment performed more accurately than both novices that had self-control and those yoked to the music experts’ schedule for two of the three Frisbee throws in retention. The experts also performed with better form than the novices that were able to self-control practice, but not those with the yoked practice.

Huet et al. (2009) found that how feedback was presented influenced the effectiveness of self-controlled concurrent feedback. A significantly greater increase in performance from the end of practice to the delayed retention period was seen for participants who self-controlled vision of a gage indicating performance but not a ghost doors condition or a yoked group. Keetch and Lee (2007) found that those in the self-controlled group significantly decreased movement time from the end of practice to delayed retention while yoked, random, and blocked groups increased movement time. However, the self-controlled group was significantly faster than only the blocked group in retention. This same pattern of results was also seen for measures of cursor error (Keetch and Lee, 2007). Similarly during delayed retention, Janelle et al. (1997) found that the self-controlled group outperformed summary, yoked, and KR groups for throwing form and accuracy (MRE). Wrisberg and Pein (2002) found that participants that viewed a model either following a self-controlled schedule or on every trial outperformed those who never viewed the model. A more autonomy supportive environment and opportunities to increase feelings of competence provide some possible explanation for positive changes in behavior discussed above, according to the SDT.

Self-controlled use of poles for assistance resulted in greater amplitudes (Wulf and Toole, 1999), longer balance time (Hartman, 2007), and better movement efficiency (Wulf et al., 2001) in delayed retention compared to yoked groups. This is an example of where participants were given an opportunity to increase experiences of competence by choosing to use the poles to assist in the performance of the task. A self-controlled viewing schedule of a model produced better form scores (Wulf et al., 2005) and self-control of the receipt of KR produced better accuracy (Chiviacowsky et al., 2008a) in comparison to yoked groups in delayed retention as well. The motivational factors in the environment of practice, including supports for autonomy and competence may have played a role in these changes in behavior.

#### Measures of how the opportunity for choice was used

Another measure of change in behavior is how participants chose to use the opportunity for choice. Those that were provided control over receipt of feedback demonstrated varied patterns of requests, but some consistent patterns emerged across studies. Janelle et al. (1997), Huet et al. (2009), and Chiviacowsky et al. (2008a) found that participants decreased requests for feedback across the acquisition period. However, Chen et al. (2002), Patterson and Carter (2010), and Hansen et al. (2011) found that the number of requests remained relatively stable across the acquisition period. In some examples, feedback requests were influenced by their performance such that participants requested feedback more often after perceived good trials than bad trials (Chiviacowsky and Wulf, 2002; Chiviacowsky et al., 2008a). Feedback requests were also influenced by previously prescribed practice schedules in a study by Patterson et al. (2011). On average, feedback requests occurred on a relatively low (<50%) number of trials with the exception of the study by Chen et al. (2002) in which participants asked for feedback on almost every trial for the duration of the acquisition period.

When participants were given control over when to receive augmented task information, Patterson and Lee (2010) found that participants also faded requests across acquisition and that requests were less frequent for easier compared to more difficult versions of the motor task. Hodges et al. (2011) found that music experts requested information more often and were the only ones to request information after relatively poorer trials. They also found that performance was more accurate when information was requested (Hodges et al., 2011). For all three studies where participants were able to request the use of poles to aid performance (Wulf and Toole, 1999; Wulf et al., 2001; Hartman, 2007) participants faded their requests for the assistive device across the acquisition period. Hartman (2007) found that participants in the self-controlled condition performed with superior balance on the no pole trials compared to pole trials, whereas the performance of those in the yoked condition was the opposite. Requests to view videos of the requisite motor action decreased across acquisition trials in studies by Wrisberg and Pein (2002) and Wulf et al. (2005). Brydges et al. (2009) found that those given outcome goals made more requests than those given process goals. Those given the opportunity to determine task difficulty by deciding racquet width gradually increased difficulty across practice, based upon the performance of previous trials (Andrieux et al., 2012). In terms of scheduling practice, some studies found evidence of schedules involving progression from easier (e.g., low fidelity or low contextual interference) to more difficult versions or schedules of the task throughout acquisition (Brydges et al., 2010; Wu and Magill, 2011). Keetch and Lee (2007) found that more switches occurred for those that practiced the easy version of the motor task compared to those that practiced the hard version of the motor task. Hodges et al. (2011) found that participants spent more time practicing the most difficult Frisbee throw.

Indicators of individual differences were also evident in the variation in total number of switches observed by Keetch and Lee (2007) and in the number of trials participants completed before choosing to stop observed by Post et al. (2011). Participants chose to switch on relatively “good” trials as indicated by faster trials preceding a switch in the study by Keetch and Lee (2007) and switches on more accurate throws in the study by Hodges et al. (2011).

Trends to decrease supports such as augmented KR or use of an assistive device across practice trials (or not) may interact with feelings of competence. Perhaps once people experience feelings of competence, they choose to decrease support. Conversely, perhaps once support is decreased learners’ feelings of competence increase. In the future the examination of how the opportunity for choice was used would benefit from predictions made within a SDT framework.

## Explanations for Changes in Behavior Observed

Throughout the motor learning literature, two main categories of explanation emerge when it comes to the differences in learning between self-controlled and yoked groups. The first of these is a series of cognitive explanations and the second are motivational explanations. Earlier research based their results on speculation, but more recently some attempts have been made to explicitly examine the mechanisms underlying the learning differences between the self-controlled and yoked conditions.

### Cognitive explanations

Janelle et al. (1995) hypothesized that the differences in the performance between self-controlled and yoked groups were because the self-controlled group processed information more efficiently and that the low frequency of feedback chosen by participants, allowed for more independent information processing. Janelle et al. (1995) also speculated that deeper information processing occurs when one is confident that they are in control over learning. Janelle et al. (1997) expanded on this explanation to include the development of better learning strategies as a possible reason for the benefits of self-control. Janelle et al. (1997) also hypothesized that the most comfortable strategy may enhance information processing.

Since then, many papers have cited increased, deeper or more efficient information processing as a possible reason for the learning differences observed between the self-control and yoked conditions (e.g., Wulf et al., 2001, 2005; Hartman, 2007; Patterson et al., 2011). Post et al. (2011) further examined this reasoning by measuring the amount of preparation time engaged in at the beginning of trials. Post et al. (2011) stated that longer preparation times paired with the better performance in retention were indicators of deeper information processing occurring in the self-controlled group compared to the yoked group.

Closely tied to the information processing explanation is the idea of cognitive effort and its possible role in the beneficial effects of self-controlled practice. Bund and Wiemeyer (2004) stated that in acquisition, self-control creates more strain on cognition, requiring decision making, monitoring and evaluating, and correction. Cognitive resources are split between learning and self-controlled processes during acquisition, however, in retention the motivational conditions and cognitive strain are equated for the self-controlled and yoked groups. Several studies discuss the importance of cognitive effort and/or investment in facilitating skill acquisition. One example of cognitive effort is discussed by Chiviacowsky and Wulf (2005) who suggested that spontaneous error estimations might contribute to the learning advantages. In another example, Patterson and Lee (2010) explain the role that an optimal amount of cognitive effort can play in expediting motor learning. In the case of a retroactive presentation of task information, the level of cognitive effort is already at a desirable difficulty so the additional cognitive processing induced by self-control did not provide additional learning benefits. However, the cognitive effort required to retrieve task information in the self-controlled proactive condition (i.e., no feedback trials) was beneficial for skill acquisition.

The results of Patterson et al. (2011) and Hansen et al. (2011) further support the idea of an optimal amount of cognitive effort in facilitating motor skill acquisition. Patterson et al. (2011) found that contrary to previous studies, some of the participants in the self-controlled condition did not choose to receive KR on perceived good trials. However those participants were still required to make a judgment on performance in order to resolve (or not) any metacognitive discrepancies between their perceived and actual motor performance, suggesting an optimal amount of cognitive effort could have still been experienced. Hansen et al. (2011) discussed how the heightened cognitive processing involved in making a choice under restrictions (i.e., pre-determined amount of trials choice was provided) emphasized perceived accuracy as underlying the KR requests. This group also avoided the processing demands for correction of poor trials which in turn strengthens the error-detection mechanism in comparison to the traditional self-controlled and yoked groups (Hansen et al., 2011). Andrieux et al. (2012) showed that even in a typical self-controlled group for example, those that adjusted task difficulty on each trial experienced a greater cognitive load compared to the yoked group. Andrieux et al. (2012) suggested the self-controlled group spent more time evaluating conditions, preparing their motor response, and interpreting the outcome of their motor response. Those in the self-controlled group were able to explore different strategies or select a strategy based upon perceived progress toward the goal. Jowett et al. (2007) hypothesized that cognitive effort ceased after reaching self-assessed proficiency which in turn prevented further learning during the remainder of acquisition trials suggesting a limitation to the extent to which self-control can elicit cognitive effort.

Referring back to Janelle et al.’s (1997) discussion of strategy, much of the cognitive effort taking place may be in the form of strategies encompassing movement, cognitive, and/or informational aspects (Bund and Wiemeyer, 2004). Building upon the discussion of strategy, Wulf and Toole (1999) predicted that if participants tried out different strategies while using the ski poles, they may have engaged in a more effective exploration of their perceptual-motor workspace with the use of the poles freeing up the cognitive resources to do so. They also explained that learners provided the opportunity to control a practice variable, arrange the environment to their own benefit. Learners in a self-controlled condition learned how to approach and learn the motor task and have the opportunity to apply strategies to enhance their metacognitive behavior (Chen et al., 2002). Wulf et al. (2001) hypothesized that self-controlled practice encourages more active engagement in the task, and strategy exploration including an optimal task solution search. A pre-determined schedule (e.g., yoked schedule) is suggested to inhibit the ability to choose, use, evaluate or change strategies creating a situation where participants could not confirm or adjust a strategy as necessary, a self-controlled practice would diminish these limitations (Wu and Magill, 2011). The self-controlled group in the study by Wu and Magill (2011) was better at confirmation and refining of strategies and they self-evaluated and changed practice based on their motor performance. The self-controlled participants used self-regulatory processes required for searching, evaluating, and choosing the correct motor solution based on feedback (Wu and Magill, 2011). In a study by Patterson and Carter (2010), learners were believed to be engaged in the metacognitive strategies required to update their decision of whether or not to receive KR. Patterson and Carter (2010) suggested that feedback on good trials confirms for participants their knowledge of the task requirements in fact coincides with the actual task requirements. In the case of multiple tasks, it is used to strengthen the inhibition of incorrect responses and further establish the link between cue and target. This strategy is used to economize invested effort. For example, Hartman (2007) showed that participants in a self-controlled condition used a pole to try out new strategies, test their effectiveness, and then modify them again on a subsequent trial.

It has also been hypothesized that self-controlled practice may increase instructional efficiency (Wrisberg and Pein, 2002) and this was elaborated upon by Chiviacowsky and Wulf (2002) who suggested that self-controlled practice is more consistent with participants’ needs. Huet et al. (2009) stated that the active role of observers benefited perception and learning, and that learners extracted perceptual information as well as information that might also guide learning. An example of how information use can be individualized is illustrated in the study by Hodges et al. (2011) where augmented information was hypothesized to play a more conformational role for novices and a more error-correcting role for music experts. In order to further examine the idea that self-controlled schedules may be more congruent with the learners needs, Chiviacowsky and Wulf (2005) manipulated this capability by preventing one group from using the information inherent in the completion of a motor action to aid in their decision of whether or not to receive KR. This group experienced degraded performance suggesting that self-control itself is not a determining factor in the success of self-controlled practice.

Arguments for both specific and general effects of self-controlled practice appear in the literature. Wulf et al. (2005) proposed that there may be additional benefits specific to the controlling a practice variable, such as the ability to extract more relevant information during observation of a model. Keetch and Lee (2007) state that learning benefits are general in nature rather than specific to the control of a particular part of practice.

Cognitive explanations have most often been discussed separately from motivational explanations, but they may be interconnected. For example, cognitive effort may also have merit as a motivational explanation. Attempts to increase the cognitive effort used during acquisition may also serve as a way to nurture an inner motivational resource though the vitalization of the learners’ sense of challenge, which can contribute to the satisfaction of the need for autonomy, a key aspect of the SDT.

### Motivational explanations

Janelle et al. (1995) proposed that those provided self-control over a practice context may experience increased confidence in their ability to perform the task. Janelle et al. (1997) expanded upon this by hypothesizing that a responsibility of reaching proficiency placed on the learner by a self-controlled practice may result in a higher motivation to perform well. Janelle et al. (1997) also suggested that it was the active involvement of the learner that resulted in motivational influences on the cognitive processes of the learner. Wulf et al. (2005) stated that a more active involvement in the learning process may lead to increased motivation, a concept echoed by Hansen et al. (2011), who suggested that increased information processing in the restricted self-control group in order to individualize practice under restrictions increased motivation to do better.

More specific to the tenets of SDT, Wulf and Toole (1999) stated that those in a yoked group had perceived control removed and therefore experienced less intrinsic motivation and invested less effort, though this was not empirically evaluated. Hartman (2007) reported that the perception of control was enough to elicit learning advantages. This conclusion was based on the lack of evidence for a beneficial effect on performance of the use of a pole over trials where no pole was used during a pilot test. Wulf et al. (2001) hypothesized that those provided with self-control over practice were more motivated to try out different strategies.

Chen et al. (2002) explained that self-initiated KR (as opposed to when participants were prompted by the experimenter) requires self-regulation and self-control and hypothesized that implicitly enhanced intrinsic motivation through self-control benefited cognitive processes. Chen et al. (2002) argued that self-regulated learners understand why they are learning and value it, which is in line with the process of internalization in the SDT. Chen et al. (2002) also hypothesized that those in a self-control group use more effective strategies, which are more comfortable and which in turn enhance information processing.

Though Andrieux et al. (2012) suggested that self-control increases self-efficacy and motivates better performance, this was not quantified in their study. Bund and Wiemeyer (2004) measured self-efficacy and concluded that self-control has positive effects on psychological states and processes as evidenced by a smaller decrease in self-efficacy perceptions after a poor trial for the self-controlled group. Bund and Wiemeyer (2004) concluded that this might result in learning because it encourages learners to try out different strategies. They also concluded that self-control results in an increased sense of self-efficacy and the option to set goals.

Some of the studies reviewed reference autonomy, though to date it has not been measured within the motor learning protocols. According to Brydges et al. (2009), increased autonomy tailors to the production of knowledge in regards to the specific needs of the learner, resulting in increased motivation suggesting that differences observed were due to the differences in how autonomy was used. According to Brydges et al. (2010), self-guided students benefited from autonomy in selection of scheduling and tailoring practice to their own needs.

Motivation has also been discussed in terms of how participants chose to control the practice variable. According to Chiviacowsky and Wulf (2002), motivational factors could underlie the preference for feedback requests after perceived good trials. Chiviacowsky and Wulf (2002) suggested it is easier to repeat a good trial than to try and correct errors from a poor trial. This difference in the required amount of effort might be a motivation to try harder for a correct response. For the yoked participants, the absence of feedback when they may have wanted it could have made the practice context less than desirable and as a result, decreased the motivation of these participants (Chiviacowsky and Wulf, 2002). This was supported by Patterson and Carter (2010) in the case of learning multiple tasks. Chiviacowsky et al. (2008a) suggested that the main benefit of self-controlled practice may be motivational. Chiviacowsky et al. (2008a) suggested that KR was chosen after perceived good trials leading to a greater “success” experience than after poor trials, which increases motivation and therefore enhances learning. Chiviacowsky and Wulf (2005) present a case of motivational factors that may contradict each other. Participants who were required to decide whether or not to receive KR about a motor response *before* that response was made may have tried harder because they requested KR, whereas the group who could request KR *after* their motor response had the opportunity to confirm a perceived good trial with a KR request. Both of these situations suggested heightened motivational factors; however results suggested that the latter of the two explanations is more likely as those who chose after a trial performed better on tests of learning.

## Measuring Satisfaction of Psychological Needs and Changes in Motivation and Behavioral Regulations

Conditions during acquisition may provide an environment conducive to the satisfaction of the need for feelings of autonomy and competence. However, to date, self-controlled motor learning protocols have not attempted to explicitly measure this. Changes in the participants’ motivation as a function of learning have not been explicitly measured within the motor learning self-controlled protocols. However, these measurements would provide a clearer explanation for behavioral changes measured as a function of self-controlled practice, rather than one based upon speculation.

One study measured feelings of self-efficacy, which is a concept similar to perceived competence and one the authors described as a “major source of intrinsic motivation” (Bund and Wiemeyer, 2004, p. 6). Bund and Wiemeyer (2004) used a custom task-specific scale, across the entire protocol, for a total of five measurements. They found that on average, participants in the self-controlled conditions reported greater self-efficacy beliefs than those in the yoked conditions. While a measure of self-efficacy gives us some insight into motivational differences between self-control and yoked conditions, these differences require further investigation.

In future experiments, the inclusion of the Intrinsic Motivation Inventory (Deci and Ryan, n.d.) would be beneficial in measuring missing steps such as motivation and regulation. The Intrinsic Motivation Inventory is used to measure, in laboratory experiments, participants’ subjective experience in relation to the task. The full version of the instrument measures seven subscales and includes 45 items (Deci and Ryan, n.d.). Many researchers have chosen to use only the subscales relevant to the research questions they were exploring, with no reported negative effects seen on the used subscales due to the removal of the others (Deci and Ryan, n.d.). The most relevant choice for the protocols currently used in motor learning studies would be the standard, 22-item version, with four subscales used in many past studies (Deci and Ryan, n.d.). The first of the four subscales is the interest/enjoyment subscale which is used to measure self-reported intrinsic motivation. The other subscales measure perceived competence, perceived choice, and pressure/tension. The value/usefulness subscale, from the original seven, could also be useful in measuring internalization. The inclusion of scales that have been validated to measure these intermediate steps in the motivation model, would clarify the currently assumed role of motivation in the self-controlled motor learning process.

Though indicators of motivation in terms of the mechanism of SDT in a learning experimental protocol have not been measured, five of the twenty-six studies have attempted to measure the motivation for the *choices* made by participants during acquisition. Chiviacowsky and Wulf (2002) were the first to use a questionnaire, post-practice, to assess when and why those in the self-controlled KR group chose (or did not choose) to receive KR during a practice period. Those in the yoked group were asked if they received KR on the correct trials and if not, when it would have been preferred (Chiviacowsky and Wulf, 2002). Since the initial use of the questionnaire, it has been used in its original form by Patterson and Carter (2010) and Patterson et al. (2011) adapted to the choice of when to use an assistive device by Hartman, 2007, and the choice of the order of the practice schedule by Wu and Magill, 2011. Chiviacowsky and Wulf (2002) found that most participants, whether they were given the choice or not, preferred to receive KR after what they perceived were good trials and not after what they perceived were poor trials. This was also true when participants were required to learn multiple versions of a task (Patterson and Carter, 2010). When Patterson et al. (2011) preceded self-controlled trials during practice with varying externally defined KR schedules; the differing schedules resulted in differential responses on the questionnaire. However, two of the three self-controlled conditions were consistent with the previous findings of Chiviacowsky and Wulf (2002) and Patterson and Carter (2010) while the third (self-controlled trials preceded by self-controlled trials) most often reported requesting feedback equally on trials perceived as good and trials perceived as poor.

Wu and Magill (2011) found that participants also preferred to switch to another task following trials perceived as good as opposed to those perceived as poor; and participants in both the self-controlled and yoked condition felt that they were able to attempt as many strategies as they wanted. In the case of requests for the use of an assistive device (pole), Hartman (2007) revealed that participants frequently asked for the assistive device when attempting a new movement strategy rather than whether the trial was perceived as either good or poor. Wulf and Toole (1999) measured feelings of security and certainty about reaching maximum amplitudes (the task goal) across practice and found that participants became less fearful of falling across practice and were uncertain about their ability to reach the task goal regardless of following a self-controlled or yoked protocol. Though some insight has been gained in terms of motivation, particularly in the case of motivation for making decisions within the practice environment, the use of valid, more specific measures of needs satisfaction, changes in quality of motivation and internalization in future research would be valuable and should be the focus of future research.

## Conclusion

The purpose of the present review was to offer a theoretical interpretation of the motor learning advantages associated with a self-controlled practice context. The tenets of the SDT proposed by Deci and Ryan (2000) offered a logical and alternative interpretation of the extant motor learning literature examining self-control. As outlined in Figure 1, the *psychological environment* and *psychological needs* of the learner are critical mechanisms facilitating early initiation of self-determined behavior. Such components as autonomy and competencies of the learner are identified as factors that subsequently impact a long term change in behavior. Within the motor learning literature, our review suggests that a self-controlled practice context is facilitating such factors as autonomy and competence of the learner, thereby supporting the psychological environment and psychological needs of the learner, leading to long term changes to a desired behavior. A desirable practice context created by attending to the psychological environment and psychological needs of the learner, subsequently leads to changes in motivation experienced by the learner. In fact, this component of the SDT is consistent with notions in the motor learning literature such that the mechanism underlying learning in a self – controlled practice context is believed to be attributed to heightened motivation to achieve the motor task goals. Finally, motor learning researchers suggest that increased motivation as a function of self-control leads to a relatively permanent change in behavior of the motor skill. This finding is consistent with the *motivational consequences* of the SDT such that increased motivation as a result of practice contexts that facilitate autonomy and competencies of the learner results in a change in behavior. Collectively, the SDT not only allows for a theoretical reinterpretation of the extant motor learning research supporting self-control as a learning variable, but also a conduit for further inquiry into understanding the mechanisms underlying learning in a self-controlled motor learning environment.

Through the vast variety of tasks, timing of protocols and variables over which control has been given, the benefit of self-control of practice to the learning of a motor task persists. These benefits are robust and present implications for teaching, coaching, and anyone responsible for organizing a practice of motor skills. Despite these findings, the question of why these benefits occur largely still remains. Two hypothesized areas of explanation may, as suggested by Bund and Wiemeyer (2004), be antagonistic in their effects. When looking at motivational reasons, the lens of SDT can help us to better understand and measure the changes occurring between the practice environment and the observed behavioral outcomes.

## Conflict of Interest Statement

The authors declare that the research was conducted in the absence of any commercial or financial relationships that could be construed as a potential conflict of interest.
